# Core-rod myopathy due to a novel mutation in BTB/POZ domain of *KBTBD13* manifesting as late onset LGMD

**DOI:** 10.1186/s40478-018-0595-0

**Published:** 2018-09-13

**Authors:** Matteo Garibaldi, Fabiana Fattori, Carlo Augusto Bortolotti, Guy Brochier, Clemence Labasse, Margherita Verardo, Emilia Servian-Morilla, Lara Gibellini, Marcello Pinti, Giulia Di Rocco, Salvatore Raffa, Elena Maria Pennisi, Enrico Silvio Bertini, Carmen Paradas, Norma Beatriz Romero, Giovanni Antonini

**Affiliations:** 1 Unit of Neuromuscular Diseases, Department of Neurology, Mental Health and Sensory Organs (NESMOS), SAPIENZA University of Rome, Sant’Andrea Hospital, Rome, Italy; 2 Unit of Neuromuscular and Neurodegenerative Diseases, Laboratory of Molecular Medicine, Department of Neurosciences, Bambino Gesù Children’s Hospital, Rome, Italy; 3 Department of Life Science, University of Modena e Reggio Emilia, Modena, Italy; 4 Neuromuscular Morphology Unit, Myology Institute, Groupe Hospitalier Universitaire La Pitié-Salpêtrière, Paris, France; 5 Neuromuscular Unit, Hospital Universitario Virgen del Rocío/Instituto de Biomedicina de Sevilla, Sevilla, Spain; 6 Department of Medical and Surgical Sciences for Children and Adults, University of Modena and Reggio Emilia, Modena, Italy; 7 Laboratory of Ultrastructural pathology, Department of Clinical and Molecular Medicine, SAPIENZA University of Rome, Sant’Andrea Hospital, Rome, Italy; 8 Unit of Neuromuscular Disorders, Neurology, San Filippo Neri Hospital, Rome, Italy

**Keywords:** Congenital myopathies, KBTBD13, Core-rod myopathy, Nemaline myopathy, Central core myopathy, LGMD

## Introduction

Few genes (*RYR1, NEB, ACTA1, CFL2*, *KBTBD13*) have been associated with core-rod congenital myopathies [7]. KBTBD13 belongs to the Kelch-repeat super-family of proteins and is implicated in the ubiquitination pathway. Dominant mutations in *KBTBD13* have been associated with a peculiar form of core-rod myopathy (NEM6) so far [10]. Childhood onset, slowly progressive proximal muscle weakness with characteristic slowness of movements and combination of nemaline rods, irregular shaped cores and unusual type2 fibres hypotrophy at muscle biopsy, were the main characteristics shared in all the affected members of the four KBTBD13 families reported in the literature [12].

We report on a 65 years old patient, of Sardinian origin, with atypical clinical and morphological presentation of NEM6 due to a novel mutation in *KBTBD13* gene.

## Methods

Patient underwent a complete clinical examination, cardiac and respiratory evaluation, biological screening for myopathies, muscle imaging studies, muscle biopsies and molecular analysis for muscle diseases.

### Morphological study

Three open muscle biopsies were obtained from deltoid, paraspinal and quadriceps muscles. Sampling site was based on neuroimaging study. For conventional histochemical techniques 8 μm thick cryostat sections were stained with haematoxylin and eosin (HE), modified Gomori trichrome (GT), Periodic acid Schiff technique (PAS), Oil red O, reduced nicotinamide adenine dinucleotide dehydrogenase-tetrazolium reductase (NADH-TR), succinic dehydrogenase (SDH), cytochrome c oxidase (COX), and adenosine triphosphatase (ATPase) preincubated at pH 9.4, 4.63, 4.35. Frozen muscle samples for immunohistochemical analysis were analysed for membrane proteins study including antibodies against dystrophin (N-ter, Rod, C-ter), sarcoglycans, α-dystroglycan (IIH6, VIA4), laminin α2, dysferlin and caveolin-3. Antibodies were visualized using innunoperoxidase techniques. Immunofluorescence study was performed for KBTBD13 with rabbit polyclonal antibodies (ab110507 abcam 1:50). Ultrastructural study by electron microscopy (EM) was performed on small muscle specimens from the last biopsy, fixed with glutaraldehyde (2.5%, pH 7.4), post fixed with osmium tetroxide (2%), dehydrated and embedded in resin. Ultra-thin sections from 6 blocks small blocks were stained with uranyl acetate and lead citrate. The grids were observed using a Philips CM120 electron microscope (80 kV; Philips Electronics NV, Eindhoven, The Netherlands) and were photo documented using a Morada camera (Soft Imaging System).

### Neuroimaging study

Whole body CT-scan and lower limb muscle MRI were obtained following a previously described protocol according to international consensus recommendation [6].

### Western blot (WB) study

Skeletal muscle of patient and age-matched control muscle specimens were isolated and snap frozen in liquid nitrogen. Tissue lysates were prepared on ice in specific buffer (50 mM Tris–HCl, pH 7.4, 150 mM NaCl, 1 mM EDTA, 1% Triton X-100 or 1% NP40, 1 mM PMSF), and protease inhibitor mixture. Lysates were cleared by centrifugation at 15000×g. Protein concentrations of the cleared lysates were determined by BCA protein assay kit (Thermo Fisher Scientific). Proteins (40 μg) were separated by SDS-PAGE, loaded in a 4–12% denaturing gel, transferred to Immobilon-P membrane and probed with rabbit polyclonal against KBTBD13 (ab110507 abcam 1:500) and beta-tubulin (sigma 1:1000). Reactive bands were detected using Lite Ablot Extend Long Lasting Chemiluminescent Substrate (Euroclone, Pero, Italy). Densitometry analysis was performed using Quantity One software (BioRad, Hercules, CA, USA).

### Molecular analysis

Next Generation Sequencing (NGS) was performed by a customized gene panel including the following 95 genes: *NEB, MYO18B, ACTA1, TPM2, TPM3, KBTBD13, KLHL40, KLHL41, LMOD3, TNNT1, CFL2, DNM2, MTM1, SPEG1, CCDC78, BIN1, RYR1, MYH2, MYH7, SEPN1, STIM1, ORAI1, PGAM2, MEGF10, MTMR14, TRIM32, FHL1, HACD1, C-term TTN, VMA21, SCN4A, CLCN1, VCP, GNE, KLHL9, HSPB8, ADSSL1, SQSTM1, MATR3, TIA1, BAG3, CRYAB, DES, FLNC, LDB3 (ZASP), HNRPDL, LMNA, FKRP, DYSF, CAPN3, FKTN, TOR1AIP1, GTDC2 (POMGNT2), GMPPB, LAMA2, COL6A1, COL6A2, COL6A3, ANO5, PYGM, CPT2, CAV3, DMD, EMD, CHKB, POMT1, POMT2, POMGNT1, FCMD, LARGE, B3GALNT2, B4GAT1 (B3GNT1), COL4A1, DAG1, ISPD, POMK (SGK196), TMEM5, SGCG, SGCA, SGCB, SGCD, DNAJB6, MYOT, TNPO3, TCAP, RAPSN, MUSK, DOK7, LAMB2, COLQ, GFPT1, CHRNE, DPAGT1, CHAT, SMN1.* Target enrichment was carried out using Nextera Rapid Capture Custom Enrichment Kit (Illumina, San Diego, California, USA) with probe design by using the Design Studio software. Paired-end sequencing was performed using an Illumina MiSeq with a sequencing depth of 100×. The Illumina VariantStudio data analysis software was used to annotate the variants. Sanger sequencing was used for *POGLUT1* gene screening and validation of *KBTBD13* variant.

### Molecular cloning

The pCMV6-AC-GFP mammalian expression vector with subcloned wild type Human KBTBD13 cDNA (kindly donated by Dr.Nyamkhishig Sambuughin of Department of Military and Emergency Medicine, Uniformed Services University, Bethesda, USA) was used as the template DNA for producing the KBTBD13G67R variant by exploiting the QuikChange XL site-directed mutagenesis kit (Agilent Technologies). Specifically, the substitution was performed by PCR amplification using the following synthetic primers carrying the desired mutation G67RF-5′-ctg cag gtg ctg cgc cgc gac cgg ccg gcg ctg-3′ and G67RR-5′-cag cgc cgg ccg gtc gcg gcg cag cac ctg cag-3′. The right insertion of the mutation was then confirmed by DNA sequencing. Plasmidic DNA was obtained by using NucleoBond Xtra (Macherey-Nagel, Duren, Germany). Then, HeLa were transfected by using Lipofectamine 3000 (ThermoFisher Scientific, Waltham, MA, USA) and plasmidic DNA (1000 ng), following manufacturer’s instructions.

### Cell culture, transfection and fluorescence microscopy

HeLa cells were cultured in Dulbecco’s Modified Eagle Medium (DMEM) supplemented with 10% foetal bovine serum (FBS) and 50 μg/ml gentamycin. Cells were maintained in 5% CO_2_ atmosphere at 37 °C. All reagents for cell culture were from ThermoFisher Scientific (Waltham, MA, USA). HeLa cells were seeded at 21 × 10^5^ cells/well in 6-well plates 24 h prior transfection, and transfected by using Lipofectamine 3000 (ThermoFisher Scientific), according to manufacturer’s instructions. Cells were incubated for 48 h, fixed with 4% paraformaldehyde (Sigma-Aldrich), and viewed by using a confocal microscopy (Leica, Wetzlar, Germany).

### Molecular modelling

The structure of KBTBD13 protein has yet to be determined experimentally but structural data are available for other BTB-containing proteins, in some cases belonging to the BTB-Kelch family. We used Homology Modelling using SWISS-MODEL [1] to predict the structure of the BTB domain only of WT KBTBD13. Since the BTB domain is known to be directly involved in the formation of Kelch proteins dimers, we calculated the putative structure of the dimer formed by the BTB domains of KBTBD13, using as template the crystal structure that of the dimeric BTB domain in SPOP:Cul3 assembly (3HQI.pdb). The structure of the G67R mutant was also calculated by Homolgy Modelling. The electrostatic potential was calculated using the APBS software [2] and the input file required to run APBS was prepared using PDB2PQR [5] on the calculated WT and mutant protein structure.

## Results

### Clinical findings

**Fig. 1:**
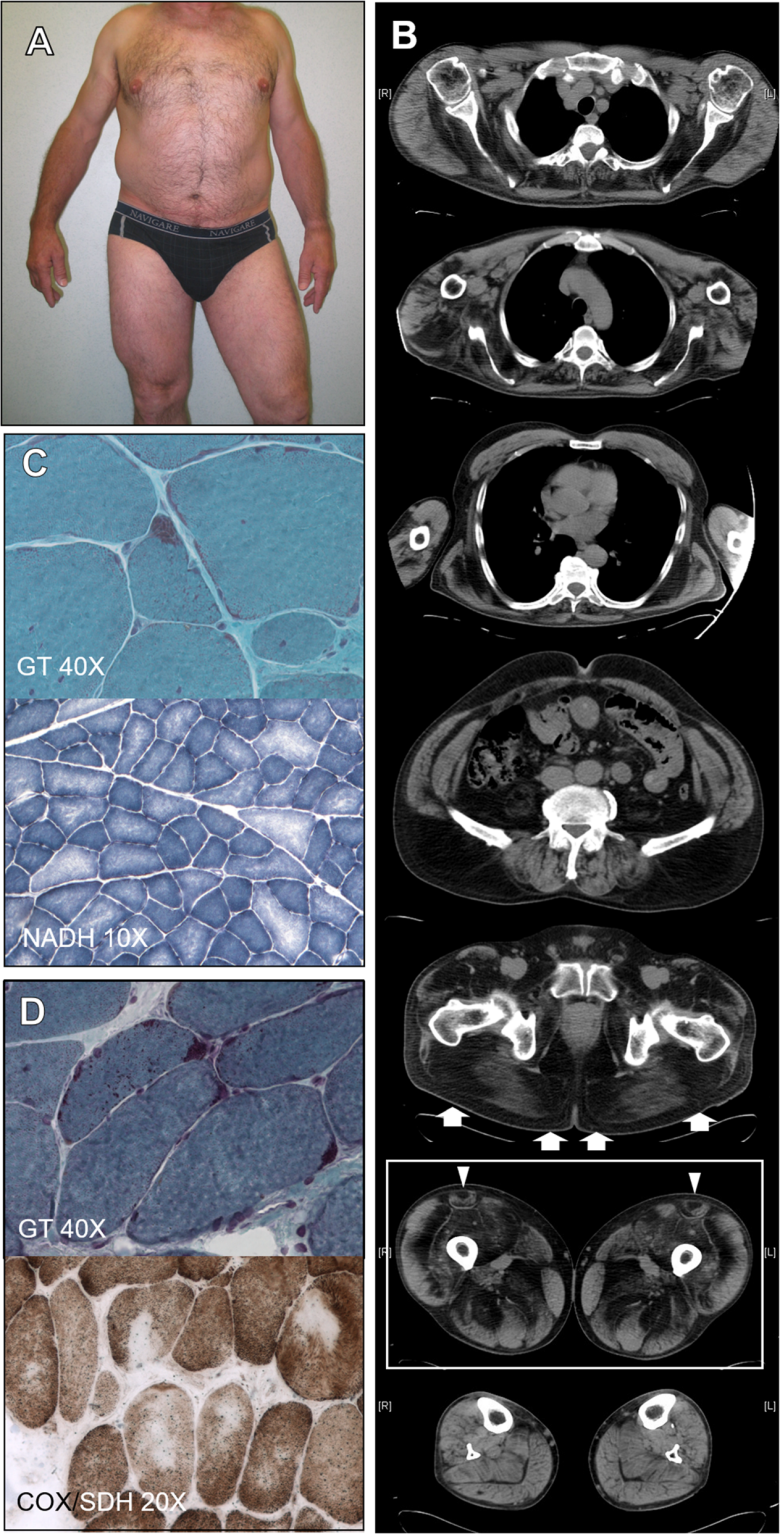
Clinical and histological findings. Picture of the patient (**a**): note the generalized muscle hypertrophy. Whole body muscle CT scan (**b**): characteristic “inside-to outside” involvement of the thighs (box); zebra pattern (arrows) of gluteus maximus; “central shadow” of rectus femoris (arrowheads); fibro-fatty replacement is also present in deltoids, subscapularis and infraspinatus, serratus, latissimus dorsi, cervical paraspinalis and rectus abdominis, iliopsoas, tensor fascia lata and gluteus maximus with while lower leg muscles are spared. Deltoid muscle biopsy at 60 years (**c**): rare cluster of subsarcolemmal rods (GT) and prevalence and hypotrophy of type1 fibres (NADH). Paraspinal muscle biopsy at 61 years (**d**): several fibres showing cytoplasmic and subsarcolemmal rods (GT) and irregular shaped areas devoid oxidative stain (COX/SDH)

The patient was born after uneventful pregnancy from healthy non-consanguineous parents. He had a 71-years-old brother with muscle hypertrophy and late onset gait disturbances who refused to be examined and tested for biological and molecular analysis. Motor milestones were normally acquired. He started to complain about slowly progressive muscular weakness of upper and lower limbs since the age of 50. First clinical examination at the age of 60 revealed generalised muscle hypertrophy, slowness of movements conferring a slow-motion bearing, proximal upper and lower limb weakness sparing distal muscles, Gowers’ sign and waddling gait (Fig. 1a). He had moderate respiratory involvement (FVC 69%) with normal cardiac evaluation. Electromyography (EMG) showed myopathic changes, and Creatine-Kinase (CK) ranged between 250 and 350 UI/L (normal values < 180 UI/L).

### Muscle imaging data

Whole body muscle CT scan performed at 60 years showed a moderate to severe fibro-fatty replacement in deltoids, subscapularis and infraspinatus, serratus, latissimus dorsi, cervical paraspinalis and rectus abdominis, iliopsoas, tensor fascia lata and gluteus maximus with sparing of leg muscles. Interestingly, several muscles presented an irregular pattern of fibro-fatty replacement. A peculiar pattern involving the internal regions and sparing the external areas (“from inside-to-outside”) was observed in thigh muscles (Fig. 1b). Moreover, rectus femoris presented the “central shadow” sign and gluteus maximus showed a “zebra” pattern of fibro-fatty replacement, characterized by consecutive strips of replaced and spared muscle tissue.

### Morphological features

**Fig. 2:**
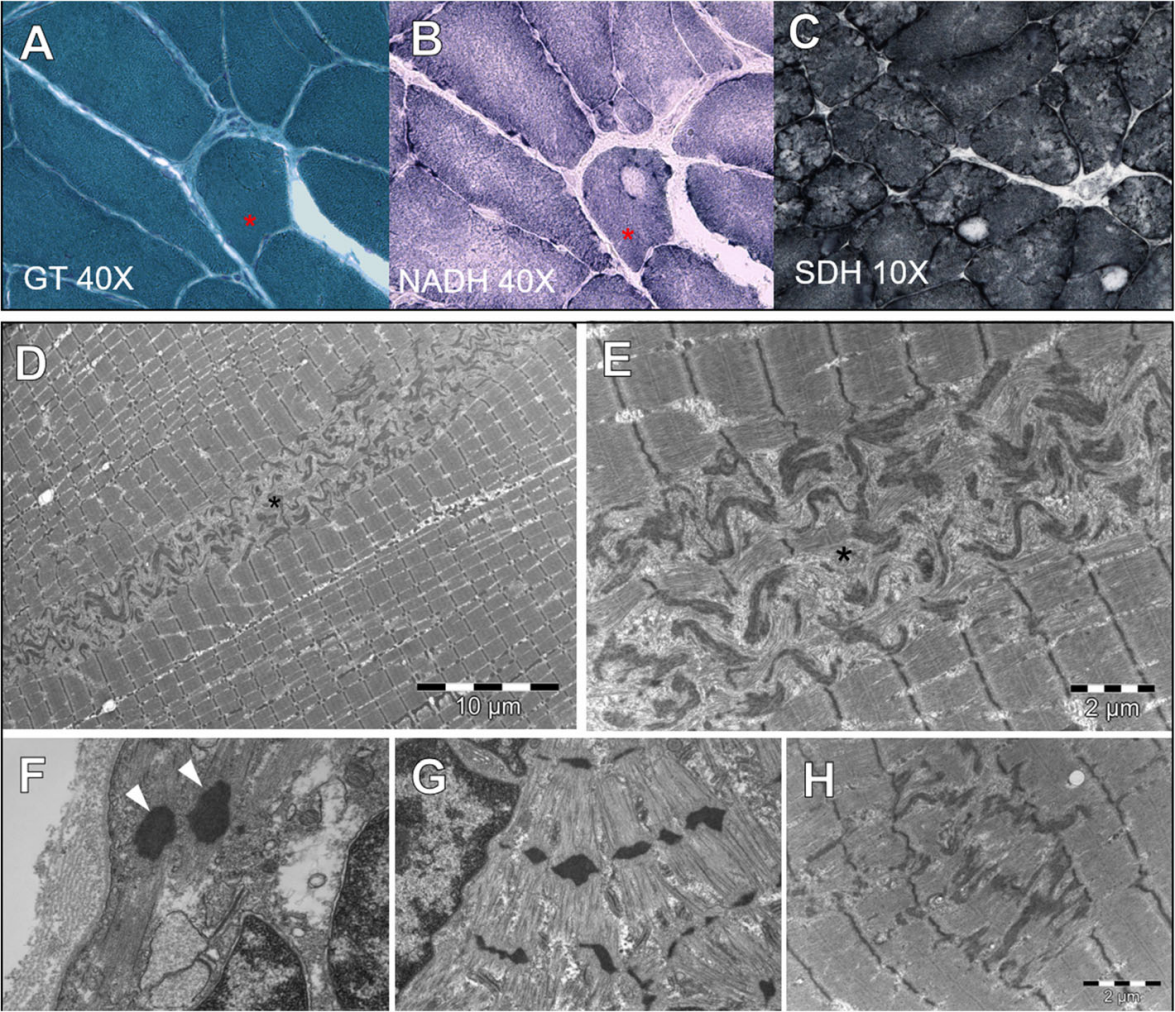
Histological and ultrastructural findings of the last muscle biopsy. Quadriceps muscle biopsy at 65 years. Sharply demarked cores in seriated sections at GT (**a**) and NADH (**b**). Cores and lobulated fibres with irregular myofibrillar network at SDH (**c**). Electron microscopy findings: cores containing abundant electrodense smeared material (**d** and **e**, asterisk); small rods (**f**,arrowheads); fragments of thickened Z-line (**g**); smaller areas of sarcomeric disorganization with Z-line smearing (**h**)

The first muscle biopsy, performed in deltoid muscle at 60 years, showed fibre size variability and splitting with type1 predominance. Small clusters of subsarcolemmal rods were detected in less than 5% of muscle fibres (Fig. 1c); immunohistochemistry for membrane proteins was normal. The second muscle biopsy was performed in paraspinal muscles at the age of 61, obtained during spinal surgery because of acute lumbar disc herniation, and showed subsarcolemmal and cytoplasmic rods in several fibres (~ 30%), type1 fibre uniformity and irregular areas of myofibrillar disorganization, sometimes resembling cores (Fig. 1d,). The third muscle biopsy performed on the quadriceps muscle at 65 years, clearly showed the presence of classic eccentric cores in some fibres (< 10%) (Fig. 2a, b, c), fibre size variability and splitting, type1 uniformity and abundant lobulated fibres with irregular myofibrillar network at oxidative stains (Fig. 2c). Ultrastructural study of the last biopsy confirmed the presence of cores with abundant electrodense longitudinally smeared material (Fig. 2d, e) and detected small nemaline rods in atrophic fibres (Fig. 2f). Areas of Z-line smearing and thickening were also observed outside cores (Fig. 2g, h). Protein aggregates were not detected.

### Molecular data

**Fig. 3:**
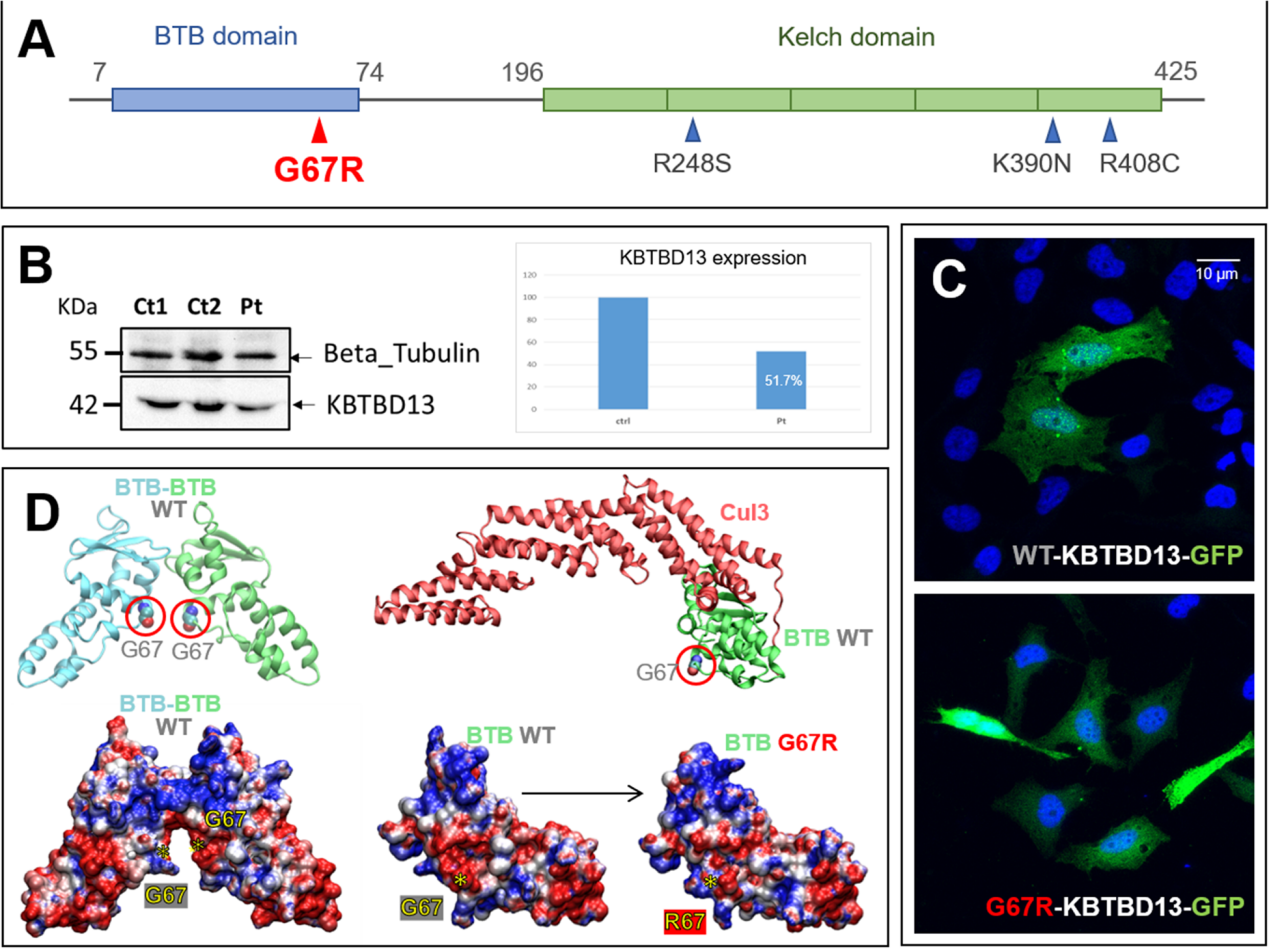
Schematic view of KBTBD13 and functional essays. Schematic view of KBTBD13 transcript (**a**) showing BTB and Kelch-repeat domains. Kelch domain is composed by five repeats. First and last aminoacidic residues of both domains are also reported. The novel G67R mutation (bold red) in BTB domain and the three previously described mutations (dark blue) in Kelch domain. WB of KBTBD13 (**b**) showing the reduced protein expression in patient’s muscle biopsy (pt) compared to healthy controls (ctrl). Representative confocal microscopy images of HeLa cells transfected with KBTBD13^WT^ and KBTBD13^G67R^ pCMV6-AC-GFP plasmids. Nuclei were counterstained with 4,6-diamidino-2-phenylindole (DAPI). Scale bars: 10 μm. No difference between wild type-KBTBD13 and G67R-KBTBD13 expression and localization in HeLa transfected cells (**c**). Molecular models (D) of WT and mutated KBTBD13. In the upper left panel, interaction of two homodimer BTB domains (light blue and green, respectively) of two KBTBD13 molecules (not shown) as obtained by molecular modelling. The protein backbone atoms are represented as ribbons, while the two Gly67 side chains are shown as Van der Waals spheres and circled in red. The upper right panel displays the calculated model of the complex between the BTB (green) and Cul3 (red). The protein backbone is represented as ribbons, and Gly67 side chain are shown as Van der Waals spheres and circled in red. The calculated model suggests that the mutated residue lies far from the interaction interface with Cul3. The lower panel on the left shows the structure of the predicted homodimer BTB-BTB (with the same size and orientation of the upper left panel) which is coloured according to its calculated surface electrostatic potential. Regions of positive and negative electrostatic potential are shown in blue and red, respectively, and the yellow asterisks indicate the position of residue 67 in both BTB domains forming the dimer. The lower panels on the right depict the changes in the surface electrostatic potential of BTB domain when Gly67 (WT) is mutated in silico into Arg67 (G67R). Note the surface electrostatic potential showing a marked loss of the surface negative potential (red colour) surrounding residue 67 in G67R mutant with respect to the WT

NGS allowed to identify a novel heterozygous c.199G > C (p.Gly67Arg) mutation in the BTB/POZ domain of *KBTBD13* gene (Fig. 3a). Other 94 genes included in the panel were ruled. The G67R variant falls in highly conserved protein domain with MAF < 0.0001 (genomAD Database) and is predicted to be pathogenetic by bioinformatic software.

### Structural and functional investigations

Transfected HeLa cells with *KBTBD13*^*G67R*^ and *KBTBD13*^*wt*^ vectors did not show any difference in cellular expression or localization of KBTBD13 (Fig. 3,C). Interestingly, we observed a reduction of 48.3% of the *KBTBD13* expression by WB on patient’s muscle biopsy compared to healthy controls (Fig. 3b). Finally, molecular modelling of KBTBD13 protein showed that residue 67 is very close to the BTB dimerization interface and the Gly-to-Arg substitution impacts on a wide area of surface electrostatic potential around the residue 67 (Fig. 3d).

## Discussion

Although some clinical features were quite similar to those previously described in KBTBD13 patients, as the characteristic slowness of movements, the late age at onset in our patient represents a novel insight for *KBTBD13*-related myopathy, since all the previously reported patients manifested a childhood onset. Moreover, our patient showed unusual LGMD-like clinical phenotype characterized by waddling gait and muscle hypertrophy. Muscle MRI findings were different from those previously reported in KBTBD13 patients and surprisingly, the peculiar “from inside-to outside” pattern of fibro-fatty replacement was very similar to what has been recently described in *POGLUT1* muscular dystrophy [13]. For this reason, we also ruled out possible mutations in *POGLUT1* gene by Sanger analysis. The “central shadow” sign of the rectus femoris has been described in *COL6*-related myopathies, but contrary to the “inside-to-outside” pattern observed in our patient, *COL6* myopathies show a typical peripheral involvement of thigh muscles [8]. Moreover, the full preservation of lower leg muscles observed in our patient could help in differential diagnosis with both COL6 and POGLUT1-related myopathies which typically show soleus involvement. To the best of our knowledge, “zebra” pattern of gluteus maximus has never been reported in other myopathies.

Concerning morphology, nemaline rods were constantly observed in all muscle biopsies performed in our patient, although sometimes detected only in few fibres or just by EM study. Only the third muscle biopsy revealed sharply demarked cores, never detected in previous reports, whereas the second one showed irregular-shaped areas devoid oxidative stains as described in previously cases. Interestingly, none of the biopsies showed the type2 fibre hypotrophy previously described in all reported KBTBD13 patients. Congenital myopathies are frequently characterised by prevalence/uniformity of type1 fibres, most of which are hypotrophic, and sometimes could manifest as Congenital Fibre Type Disproportion (CFTD), characterised by marked hypotrophy of all type1 fibres mixed with normal type2 fibres [4]. Conversely, type 2 hypotrophy represent an uncommon finding among congenital myopathies, and more broadly among inherited myopathies. Type2 atrophy is frequently observed in acquired conditions as cachexia, aging or corticosteroid-induced myopathy [3], and among inherited myopathies it has been rarely reported [9]. Among congenital myopathies and particularly nemaline myopathies, the type2 hypotrophy has been described only in KBTBD13 and represented a specific signature of NEM6. Conversely, our patient showed type1 prevalence or uniformity, as commonly observed in many congenital myopathies.

The clinical and histological variability observed in our patient compared to the previously reported NEM6 patients, could be related to the different localization of the *KBTBD13* mutation. In fact, all the three mutations previously reported are located in Kelch domain, whereas G67R mutation detected in our patient is in the BTB/POZ domain. Possibly, the surprising reduction of KBTBD13 protein by WB analysis might be explained considering the effect of G67R mutation. KBTBD13 proteins assemble into dimers through self-association of the BTB domains. Each BTB domain interacts with the N-terminus of one Cul3 molecule forming a RING ubiquitin ligase (Cul3-RL) complex capable of ubiquitination [11]. Our molecular modelling revealed that location of Gly67 is very close to the BTB dimerization interface; on the contrary, the location of residue 67 is rather far from Cul3-binding interface. Since the mutation described concerns the substitution of a small, hydrophobic amino acid (Gly) with another featuring a rather bulky side chain, positively charged at physiological pH (Arg), it is likely that the mutation alters the surface properties of the protein, especially the surface electrostatic potential. Electrostatic interactions across protein interfaces are known to be crucial to the formation of multimers and complexes that often represent the biologically active conformation of proteins. Our computational investigations also suggest that the G67R mutation alters the surface electrostatic potential, probably impacting the tendency of BTB dimerization in vivo. Nevertheless, this study concerns only one patient and more evidences are needed to conclusively state the effect of mutations in BTB/POZ domain of *KBTBD13* gene.

## Conclusions

In conclusion our findings broaden the clinical, histological and genetical spectrum of the ultra-rare *KBTBD13*-related myopathy and enlarge pathophysiological knowledges about nemaline myopathies, showing that NEM6 can have an adult onset, may show muscle hypertrophy with peculiar “inside-to-outside” MRI/CT pattern, and may lack of type2-hypotrophy at muscle biopsy. Different domains involved of *KBTBD13* could lead to the phenotypic variability in NEM6.
